# Metagenomic Analysis of Microbial Alliances for Efficient Degradation of PHE: Microbial Community Structure and Reconstruction of Metabolic Network

**DOI:** 10.3390/ijerph191912039

**Published:** 2022-09-23

**Authors:** Pan Xu, Xiaoxiao Chen, Kai Li, Rong Meng, Yuewu Pu

**Affiliations:** 1 School of Biology and Biological Engineering, South China University of Technology, Guangzhou 510006, China; 2 The Husbandry Technology Promotion Center of Inner Mongolia, Hohhot 010051, China

**Keywords:** phenanthrene degradation, metagenomics analysis, microbial community structure, reconstructing metabolic network

## Abstract

Polycyclic aromatic hydrocarbons are a widespread organic pollutant worldwide. In this study, a highly efficient phenanthrene (PHE)-degrading microbial community was enriched from oil extraction soil, which could degrade 500 mg/L PHE within 4 days. Using 16S rRNA sequencing, the dominant bacteria in this community at the phylum level were found to be *Proteobacteria*, *Actinobacteria*, and *Firmicutes*. Metagenomic annotation of genes revealed the metabolic pathways and the contribution of different bacteria to the degradation process. *Pseudomonadaceae* contributed multiple functional genes in the degradation process. This study revealed the functional genes, metabolic pathways, and microbial interactions of the microbial community, which are expected to provide guidance for practical management.

## 1. Introduction

Polycyclic aromatic hydrocarbons (PAHs) are aromatic compounds with two or more PAHs in their configuration, which are persistent organic pollutants widely existing in the environment [1]. PAHs are carcinogenic, mutagenic, and teratogenic to animal and human health [2,3] and easily accumulate in human tissue [4], which is currently a worldwide environmental problem [5,6]. PAHs are nonpolar, hydrophobic, and lipophilic. Their complex aromatic structure and limited bioavailability make them chemically stable and resistant to microbial degradation [7], which allows PAHs to remain persistent and stable in the environment. PAHs originate from a wide range of sources, including natural (such as biological generation) [8] and human sources. The exploitation, transportation, and use of petroleum make a significant contribution, and the concentration of PAHs in petroleum exploitation areas is considerably higher than in other places [9]. Phenanthrene (PHE) is a PAH that is frequently detected in the environment [10]. It is widely distributed and has high toxic potential; therefore, it must be removed from the environment [11]. Consequently, PHE is frequently used as a model compound to study PAHs [12,13].

Certain physical or chemical methods, such as incineration, UV oxidation, and solvent extraction, have disadvantages such as high cost, complex treatment, difficult supervision, and failure to completely destroy PAHs, whereas biological methods are considered to be efficient and environmentally friendly remediation technology [14,15]. Multiple pure bacteria have been screened to degrade PAHs [16], including Pseudomonas stutzeri, Bacillus simplex, and Bacillus pumilus [13]. However, bacterial consortia have been reported to show superior degradation efficiencies [17], because they can degrade PAHs through complex co-metabolic phenomena [15].

Coordination between bacterial alliances is very important and complicated in the process of explaining PAHs. Because many microorganisms cannot be cultured and the tools of molecular biology are limited, it is difficult to observe microbial interactions in real time and in situ. Therefore, more omics tools are needed such as macro genomics to rebuild the microbial degradation of PAH coexistence microbial interaction networks [18] to help understand the principle of microbial degradation. Metagenomics, the study of sequencing DNA collected from the environment, is useful for determining microbial diversity and function. Metagenomics can help to identify new genes and metabolic pathways, study new genes related to pollution metabolism [19], and provide key information on metabolism and functional variation.

However, there have only been few reports to date focusing on the interactions between PHE degrading bacterial communities and their contribution to metabolic pathways. Therefore, a study was devised with the objective of developing new stable enriched mixed bacterial cultures from oil fields that are capable of degrading PHE. We studied the effects of different concentrations of PAHs in the presence of degradation efficiency. The microorganisms were investigated by means of 16S rRNA sequencing at different PAH concentrations and the bacterial community succession of time. This study obtained the abundance information of functional genes and functional microorganisms, as well as learned about the PHE degradation pathway and mechanism. Furthermore, a metabolic network was reconstructed, and functional genes at different stages of the degradation pathway were associated with microorganisms to clarify the cooperative relationship between them.

## 2. Materials and Methods

### 2.1. Soil Sampling and Enrichment

Soil samples (0–10 cm from the surface) were collected from the Karamay oil field (Xinjiang, China). A mineral salt medium (MSM; Supplementary Table S1) with PHE (Aladdin) as the sole carbon source was used to enrich the PHE-degrading microbial community. The enrichment process was as follows: PHE was dissolved in acetone to prepare a PHE concentrate (5 g/L). PHE concentrate (0.4 mL) was added to sterilized MSM, and a 20 mL–100 mg/L mineral salt–PHE medium was prepared after acetone was volatilized. Then, 1 g of soil sample was added; the cone-shaped flask was transferred to a shaking incubator (200 rpm) and incubated at 30 °C for 3 days. The culture (2 mL) was transferred to a 20 mL mineral salt–PHE medium with a PHE concentration of 200 mg/L, and cultured for 3 days. Finally, the culture was transferred to a mineral salt–PHE medium with a PHE concentration of 300 mg/L, and the bacterial community for PHE degradation was obtained under the same conditions for 3 days.

### 2.2. PHE Degradation Experiment

The microbial community was inoculated in SOB medium (Sangon Biotech, Shanghai, China) and shaken at 30 °C for 24 h. The culture medium was centrifuged at 2652× *g* for 5 min, washed with MSM three times, and then resuspended in MSM.

In the degradation test, the initial concentrations of PHE were 100 mg/L, 250 mg/L, and 500 mg/L. Resuspension inoculants (2 mL, OD600 = 1) were inoculated in 20 mL of mineral salt–PHE medium. A control group was set up to assess abiotic loss. All experiments were performed in triplicate and destructively sampled 2, 3, 4, 5, and 6 days later; then, the biodegradation of PHE by the microbial community was evaluated. In addition, samples taken on the third and sixth days were refrigerated at −80 °C for subsequent high-throughput sequencing. The samples on the third and sixth days of each concentration were named F100.3, F250.3, F500.3, F100.6, F250.6, and F500.6, respectively.

### 2.3. Extraction and Detection of PHE

The sample was extracted with n-hexane at the same volume as the culture medium, and the process was repeated twice. PHE concentration was determined using high-performance liquid chromatography (LC-20AT, SHIMADZU, Kyoto, Japan). The mobile phase consisted of acetonitrile and water at a ratio of 9:1 (*v*/*v*). A C18 column (250 mm × 4.6 mm) was used, 20 µL of each sample was injected, and the UV detector was set at 254 nm. To calculate the PHE concentration, a standard curve of PHE external standard detection was constructed with a correlation coefficient of 0.99.

### 2.4. Illumina MiSeq Sequencing of Microbial Community and Analysis

Culture medium was subjected to 16S rRNA sequencing and metagenomic analysis on the third and sixth days of degradation of PHE medium at the above three concentrations (a total of six samples, named F100.3, F250.3, F500.3, F100.6, F250.6, and F500.6). Here, 100, 250, and 500 represent the PHE concentrations, and 3 and 6 represent the time of cultivation (i.e., the third and sixth days, respectively). The forward and reverse primers used for 16S rRNA analysis were Bakt_341F (CCTAYGGGRBGCASCAG) and Bakt_806R (GGACTACNNGGGTATCTAAT), respectively. Metagenomic analysis and 16S rRNA sequencing were performed by Novogene (Beijing, China). DNA was analyzed by NovaSeq6000, and effective tags were obtained after data processing. Sequences were clustered into operational taxonomic units (OTUs) with 97% identity by Uparse software (Uparse V7.0.1001, http://www.drive5.com/uparse/, accessed on 21 May 2022) [20], and species annotation analysis was performed using the Mothur method and SSUrRNA database [21] of SILVA132 (http://www.arb-silva.de/, accessed on 21 May 2022) [22] (threshold was set at 0.8–1). Then, Qiime software (Version 1.9.1) was used to calculate the alpha diversity index. The functional genes related to the PHE degradation pathway were annotated and mapped to the Kyoto Encyclopedia of Genes and Genomes (KEGG, Version: 2018.01) [23,24], while functions of orthologous groups were annotated to the evolutionary genealogy of genes, non-supervised orthologous groups (eggNOG, Version: 4.5) [25], and the function and relative abundance of carbohydrate enzymes were annotated to the Carbohydrate-Active Enzymes Database (CAZy, Version: 2018.01) [26].

## 3. Results and Discussion

### 3.1. The Degradation of PHE

**Figure 1:**
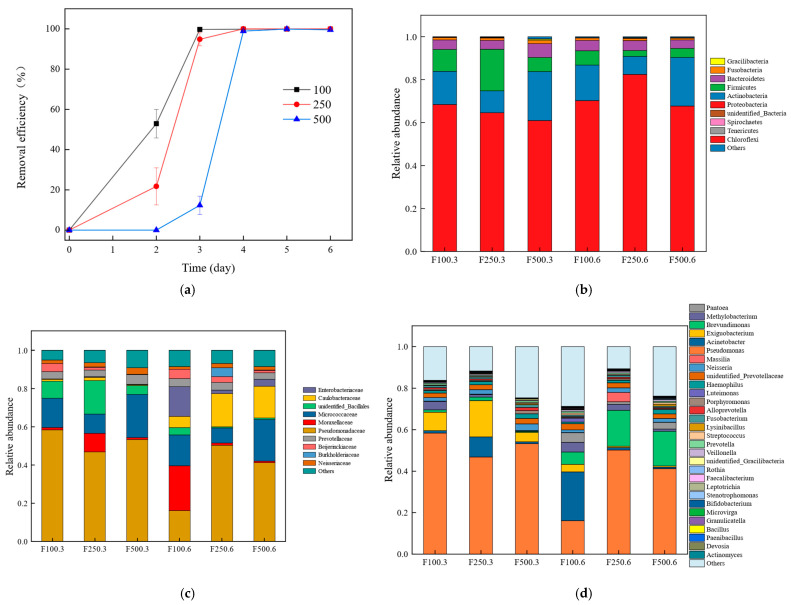
The removal efficiency of PHE (**a**); structural distribution of bacterial community at phylum (**b**), family (**c**), and genus (**d**) levels.

The removal efficiency of PHE is shown in Figure 1a. On the second day, the removal efficiencies of 100 mg/L and 250 mg/L PHE reached 52.90% and 21.76%, respectively. However, 500 mg/L PHE did not degrade, which may be attributed to the inhibitory effect of higher PAH concentrations on microbial cell growth [27]. Other studies have shown that compounds at high concentrations inhibit or reduce the activity responsible for their metabolism [28], which may also explain why 500 mg/L of PHE did not degrade. On the third day, the removal efficiencies of 100 mg/L and 250 mg/L PHE were 99.72% and 94.84%, respectively. At this time, a lag phase of 500 mg/L PHE passed, and its removal efficiency reached 12.33%. On the fourth day, 250 mg/L PHE was degraded, and 500 mg/L PHE removal efficiency increased to 98.90%. The reason for the sudden increase in removal efficiency may be that the intermediate metabolites of the third day could be used to promote the growth of some bacteria that cannot directly use PHE, and these bacteria cooperated with other bacteria to promote the degradation of PHE, while the metabolites produced could further promote the degradation of PHE, forming a virtuous cycle. It was reported that the selected microbial consortium could degrade over 96% of 200 mg/L PHE on the third day [29], and certain researchers used a single strain to degrade 400 mg/L PHE at a rate of 89.5% after 7 days [30]. Compared with the other studies mentioned above, the microbial community in this study had a significant advantage in its ability to degrade PHE.

### 3.2. Microbial Community Structure of the Six Samples

Alpha diversity was used to analyze microbial community diversity within the samples. Microbial community diversity and richness were measured using Shannon, Simpson, chao 1, ACE, and coverage indices (Supplementary Table S2). The coverage index was ≥0.999, indicating that the sequencing results could represent the actual situation of microorganisms in the samples. Among the six samples, the number of operational taxonomic units of F500.6 was the largest. In general, a higher concentration of PHE led to lower diversity, as well as lower metabolic and phylogenetic diversity [31]. However, in this study, the trend of community structure richness was the opposite, which may be because this microbial community had a higher degradation potential. This result also supported to some extent the conclusion in Section 3.1 that some bacteria use intermediate metabolites to grow. The degradation trend of PHE was consistent with the results of community diversity and richness, indicating that the PHE degradation level was positively correlated with bacterial abundance, and that the cooperation between strains led to more efficient PAH degradation [32].

Figure 1b shows the relative abundance of the six samples at phylum levels (abundance of top 10). The major phylum in the samples were *Proteobacteria*, *Actinobacteria*, *Firmicutes*, *Bacteroidetes*, and *Fusobacteria*. The relative abundance of the *Proteobacteria* in the six samples was 68.43% (F100.3), 64.54% (F250.3), 60.94% (F500.3), 70.19% (F100.6), 82.40% (F250.6), and 67.68% (F500.6). *Proteobacteria* have been reported as potential PAH-degrading bacteria [33]. On the third day, the relative abundance of the *Proteobacteria* was higher in the samples with higher removal efficiency. In addition, there were different degrees of *Proteobacteria* accumulation in the samples on the sixth day compared with that of the samples on the third day. The results were similar to those reported by others [34]. Other studies have shown that reduced *Proteobacteria* abundance leads to reduced biodegradation of PAHs [35]. Therefore, the high abundance of *Proteobacteria* may be responsible for the high degradation efficiency of PHE in this bacterial community. Certain bacteria in the phylum *Actinobacteria*, such as *Rhodococcus* sp. P18 and *Gordonia* sp. H19, can relatively efficiently degrade high-molecular-weight PAHs [36]. Studies have shown that *Firmicutes* and *Bacteroidetes* are common phyla isolated from PAH-enriched cultures [37], and *Bacteroidetes* members are particularly proficient at degrading various polymers [38].

As shown in Figure 1c, *Pseudomonadaceae*, *Moraxellaceae*, *Micrococcaceae*, and *unidentified_Bacillales* were the dominant families in all samples. In addition to F100.6, the relative abundance of *Pseudomonadaceae* in the other five samples was >40%, and it is also a bacterium that has been reported to degrade and detoxify PAHs [39]. Similar to the results of other studies [40], in this study, *Micrococcaceae* (7.98–21.88%) was a relatively abundant family of PHE-degrading bacteria. On the third day, the relative abundance of *Caulobacteraceae* was 1.12% (F100.3), 1.68% (F250.3), and 0.46% (F500.3). After 3 days of enrichment, its relative abundance increased to 5.95% (F100.6), 17.32% (F250.6), and 16.68% (F500.6). *Caulobacteraceae* can be found in oil-contaminated soils and can tolerate heavy metals [41]. Some bacteria in the *Bacillales* family may play a pioneering role in microbial recovery by consuming mobilized organic matter even under adverse environmental conditions [42].

Figure 1d shows the distribution of the bacterial community at the genus level (abundance of the top 30). Overall, the relative abundance of *Pseudomonas* was highest among the six samples. According to a previous report, *Pseudomonas* effectively biodegraded PAHs and heterocyclic derivatives via lateral dioxygenation pathways [43]. In addition, *Pseudomonas* can employ biosurfactants [44], resist heavy metals [45], and degrade high-molecular-weight PAHs [46]. This may be the basis for efficient degradation of PHE. Interestingly, the trend of the relative abundance of *Exiguobacteriu* increased and then decreased. In contrast, *Brevundimonas* accumulated to varying degrees on the sixth day. The literature suggests that *Exiguobacterium* can effectively degrade PHE [47] and enhance the removal of crude oil [48]. In addition, studies found the same trend in the late removal of PAHs from compost [49]; therefore, it may be that PHE was degraded on the sixth day, leading to a decline in its abundance. The major microbial communities in this study were associated with the degradation of PHE and PAHs, which could facilitate PHE removal.

### 3.3. Metabolic Analysis of Evolutionary Genealogy of Genes: Non-Supervised Orthologous Groups Database (eggNOG)

**Figure 2:**
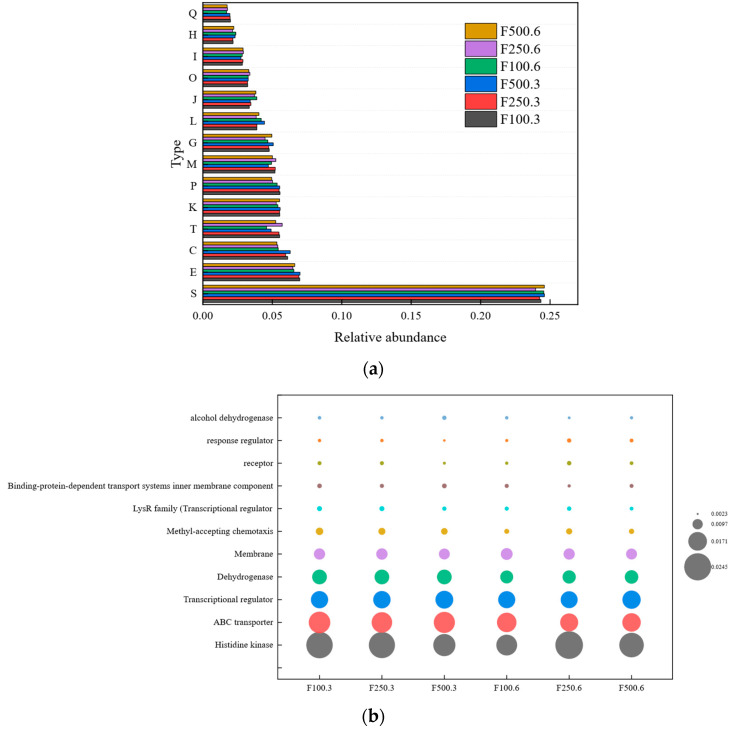
The relative abundance of eggNOG at level 1 (**a**) and level 2 (**b**).

Figure 2a (showing abundance of top 14, the specific meaning of the abbreviations in Figure 2a is given in Supplementary Table S3) shows the gene function annotation and the relative abundance of gene number of the eggNOG level 1 database. The results show that amino-acid transport and metabolism (6.47–6.99%), energy production (5.32–6.28%), and conversion and signal transduction mechanisms (4.57–5.71%) were the three most abundant. PAHs can cause genetic aberrations [14]; therefore, the relative abundance of replication, recombination, and repair was also high; in the six samples, the relative abundance was 3.88% (F100.3), 3.88% (F250.3), 4.43% (F500.3), 4.19% (F100.6), 3.83% (F250.6), and 4.02% (F500.6). This function can repair genes that allow microbial communities to survive and then use and degrade PHE. Biosurfactants are generally produced from secondary metabolites of organisms [50], which can increase the solubility of hydrophobic compounds and the bioavailability and biodegradability of hydrophobic compounds such as polycyclic aromatic PHE. Glycolipids and lipopeptides are biosurfactants frequently secreted by microorganisms in the presence of aromatic compounds. In the eggNOG database comments, secondary metabolites biosynthesis, transport, and catabolism were annotated, and their relative abundance in six samples ranged from 1.70% to 1.97%. Moreover, emulsification was observed during the extraction of residual PHE.

As shown in Figure 2b, a further level 2 data study on eggNOG showed that histidine kinase, ABC transporter, transcriptional regulator, dehydrogenase, and membrane were functions of the gene with the highest relative content (relative abundance >1%). According to previous reports, histidine kinase may mediate signal transduction in bacterial chemotaxis [51]. In addition, the LysR family (transcriptional regulator) may regulate genes involved in the catabolic metabolism of aromatic compounds, cell movement, and quorum sensing [52], which can drive bacteria to move to a better ecological niche, adhere to the oil–water interface, have better contact with PHE, and promote its degradation. ABC transporters (ATP-binding cassette transporters) with a relative abundance of approximately 1.61–1.93% can be used to transport substrates [53], such as some carbohydrates, amino acids, ions, peptides, hydrocarbons, and other molecules, and the abundance of ABC transporters in the annotated results may indicate that exposure to PHE at different concentrations led to changes in some metabolic processes, material transport, and nutrient absorption. Alcohol dehydrogenase (approximately 0.27–0.39%) is responsible for a series of reactions that transfer intermediate metabolites resulting from cyclic cleavage of aromatic compounds to protocatechuate, which is then degraded by the central aromatic process [54]. In summary, the eggNOG database was used to annotate the functions of several genes that promote the conversion and degradation of aromatic compounds, also representing the mechanism for the efficient degradation of PHE in this microbial community.

### 3.4. Metagenomic Analysis of Carbohydrate-Active Enzymes (CAZy)

**Figure 3:**
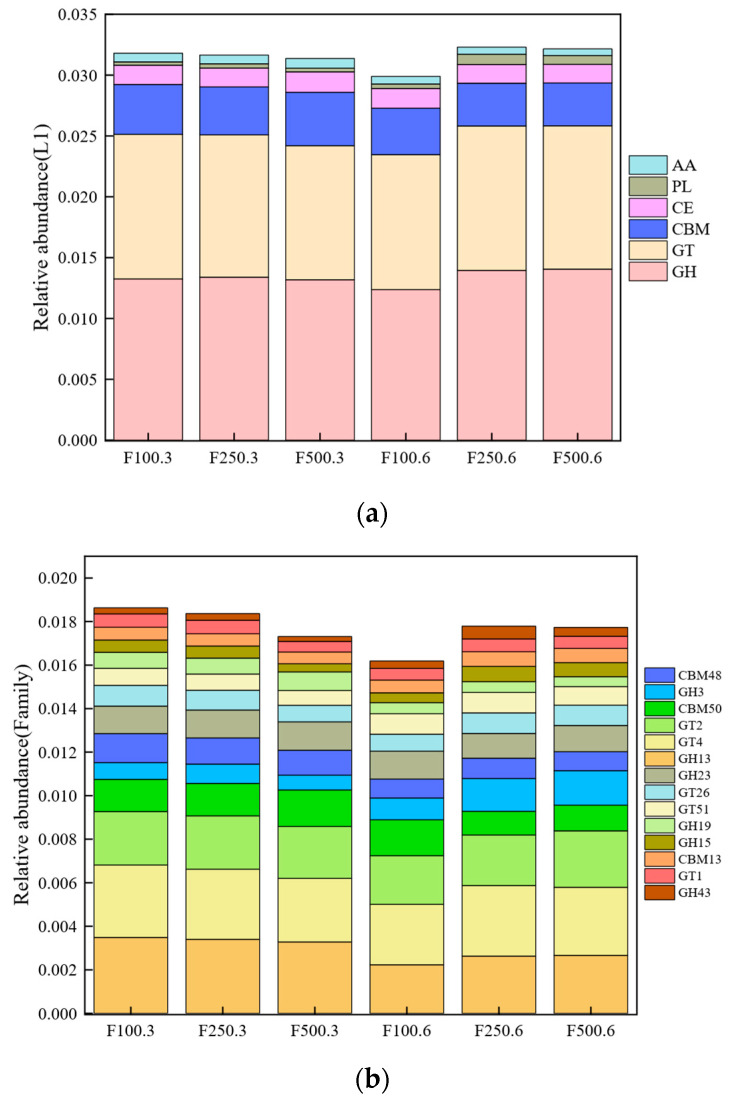
Relative abundance of the CAZymes at the L1 (**a**) and family (**b**) levels.

CAZys can degrade, modify, and form glycosidic bonds, along with their conversion and energy acquisition, and carbohydrate enzymes are essential for catalyzing hydrocarbon absorption [55]. They can be divided into six categories: carbohydrate-binding modules (CBMs), carbohydrate esterases, glycoside hydrolases (GHs), glycosyl transferases (GTs), polysaccharide lyases, and auxiliary activities. Certain CAZys can react directly with PAH contaminants, oxidize phenolic and nonphenolic aromatic compounds, and effectively oxidize PAHs with three or four aromatic rings [56]. As shown in Figure 3a, GH and GT were the dominant enzymes in all six samples. Certain GH and GT enzymes function in cutting and cracking polymers [57]. CBMs have also been widely found. CBMs can promote the interaction between a given enzyme and its substrate, thereby improving its catalytic efficiency [58]. It is speculated that CBMs may be more beneficial for targeting the enzyme to PHE or some other intermediate metabolite or binding the active site of the enzyme to PHE or some other intermediate metabolite, thus being more likely to cause damage and degradation of PHE.

To better understand the function of CAZys in the microbial community, further analysis was conducted at the family level, as shown in Figure 3b. GH13, GT4, GT2, CBM50, GH3, and CBM48 were the main enzyme types. Some modules, such as CBM48, are frequently present in the GH13 family structure, leading to amylase targeting of starch and binding [59], which may support our conjecture above. Some GH13 subfamily enzymes, such as alpha-amylase, directly degrade alkanes and are highly adaptable to extreme environments. The GH3 family may be associated with hemicellulose degradation; GT2 and GT4 may be disaccharides associated with the synthesis of oligosaccharides, polysaccharides, CBM50, and cellulose. Furthermore, lignin can be a very good combination of mutual coordination between enzymes, which can lead to the degradation of lignin and cellulose [60], not just because PHE and polysaccharide materials such as lignin have similar structures [61], indicating that CAZys may participate in the degradation of PHE. Moreover, multiple studies have shown that the biodegradation of PAHs can be greatly promoted along with the degradation process of lignin, cellulose, and other polysaccharides, such as the promotion of PAH degradation by compost [62] and the degradation of PAHs by some white rot fungi [63]. The extracellular ligninolytic enzyme system of fungi is directly related to the degradation and detoxification of organic pollutants, and even promotes the degradation of organic pollutants [56], as well as a wide range of substrate specificities, so that they can transform multiple substrates, even those that are difficult to degrade. In conclusion, the synergy among different CAZys was beneficial for PHE removal in this study.

### 3.5. Metagenomic Analysis of Kyoto Encyclopedia of Genes and Genomes (KEGG)

To further study the functional composition of this microbial community and the metabolic mechanism of PHE, the genes were annotated using the KEGG database. First, KEGG level 1 data were analyzed (Supplementary Figure S2). Metabolism was the most abundant, and the relative abundances of the six samples were 17.74% (F100.3), 17.69% (F250.3), 18.07% (F500.3), 17.80% (F100.6), 17.49% (F250.6), and 17.78% (F500.6). The next most abundant was environmental information processing (6.29–7.23%), followed by cellular processes (4.43–4.97%), genetic information processing (3.91–4.31%), human diseases (2.06–2.28%), and organismal systems (0.96–1.02%).

Analysis of the KEGG secondary annotation results showed that carbohydrate metabolism (4.67–4.95%), amino-acid metabolism (4.56–4.82%), signal transduction (2.86–3.41%), energy metabolism (3.09–3.3.35%), and membrane transport (2.77–3.26) were the top five gene sequence functions in the relative abundance of the six samples (Supplementary Figure S3). Carbohydrate metabolism and amino-acid metabolism are related to the degradation of PAHs, and enrichment regulation of carbohydrate metabolism genes is helpful to enrich the genes of degrading PAHs [64]. Amino acids in all the various microbial cell metabolisms play a central role, such as glutamic acid, which is a key metabolite that participates in protein synthesis and other basic processes, such as the citrate cycle, which can help to link nitrogen and carbon metabolism, playing an important role in various harmful environmental stresses [65]. Moreover, amino-acid metabolism is strongly correlated with PAH degradation, and the presence of PAHs can upregulate genes related to carbohydrate and amino-acid metabolism [66]. Lipid metabolism is also associated with PAH metabolism [67]. Several aromatic substances and PAHs can force bacteria to produce lipids [68], which can often be a biosurfactant that increases the solubility and bioavailability of PAHs, thereby promoting their degradation. Consistent with the annotation results of eggNOG, gene replication and repair were also annotated in the KEGG database, as well as folding, sorting, and degradation, which can maintain the homeostasis of the organism by removing defective proteins [69]. In addition, cell motility was annotated to support the results of Lys family and chemotaxis in eggNOG, indicating that, under PHE stress, bacteria can indeed move to the ecological niche conducive to their survival, such as the junction between biological surfactant and water, i.e., the water–oil interface. More importantly, the abundance of xenobiotic biodegradation and metabolism (0.815–1.11%) was also relatively high, which indicated that the bacterial community concentrated a large part of its strength on the degradation of xenobiotics, such as the degradation of PHE.

**Figure 4:**
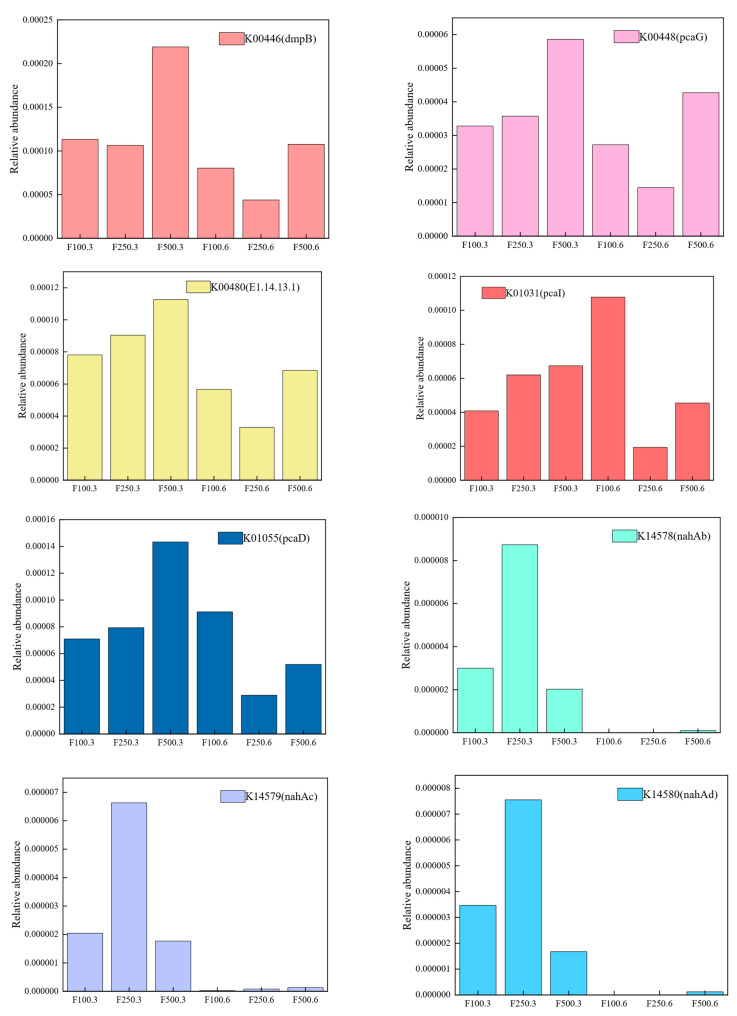

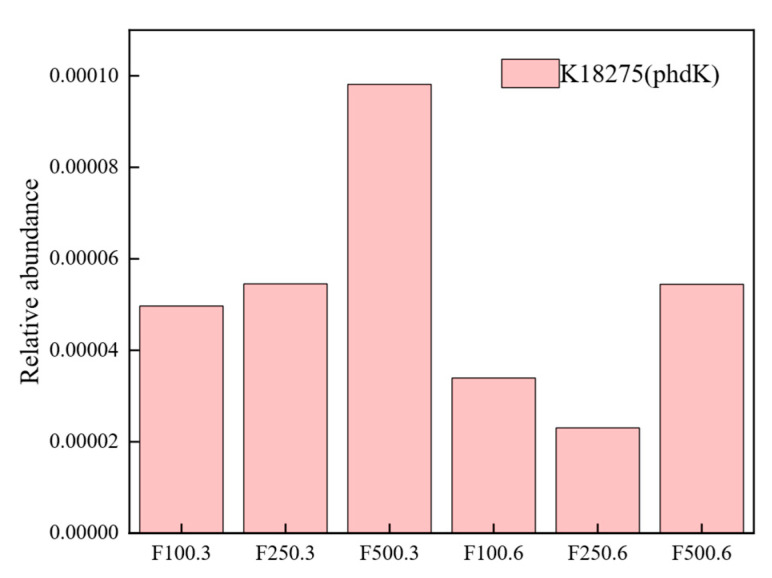
Relative abundance of some functional genes in the PHE degradation pathway.

**Figure 5:**
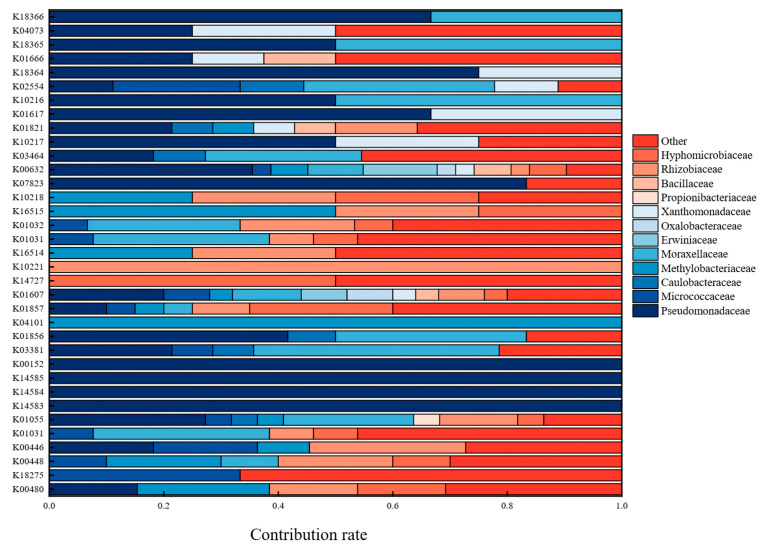
The contribution of bacteria (family) to PHE degradation pathway.

The metabolic pathway of PHE was reconstructed according to the function of the KEGG-annotated genes (Supplementary Figure S4). The microbial community degrades PHE via two pathways: the naphthalene and salicylate pathway and the phthalate and protocatechuate pathway. The final metabolites enter the citrate cycle (TCA cycle) and glycolysis/gluconeogenesis. This proved that the microbial community could mineralize PHE, and the final metabolites were water and carbon dioxide. The TCA cycle is essential for various physiological functions of microorganisms, and multiple metabolites produced in the cycle are involved in cellular respiration, protein translation, cell proliferation, genome maintenance, and other processes [70]. Glycolysis converts glucose into pyruvate and a small amount of ATP (energy) and NADH (reducing power), which are important central metabolic pathways. Interestingly, not many genes were annotated in the upstream pathway of phenanthrene degradation, including PAH dioxygenase large subunit and phenanthrene 1,2-monooxygenase, which initially lead to PHE ring opening. This may be because the KEGG database only contained the nahAc and nidA genes [71], which were from *Pseudomonas* and *Mycobacterium*. Instead, it was noted that naphthalene 1,2-dioxygenase subunit alpha (K14579, K14580, K14578, as shown in Figure 4), an enzyme encoded by this gene, is said to have a wide range of substrate specificities, which can enable the oxidative ring opening of some PAHs, such as pyrene and fluorene opening [72]. Moreover, genes that entered the naphthalene and salicylate and phthalate and protocatechuate pathways were annotated and coded for salicylate hydroxylase (K00480), 2-formylbenzoate dehydrogenase (K18275), protocatechuate 3,4-dioxygenase, alpha subunit (K00448), protocatechuate 4,5-dioxygenase, alpha chain (K04101), catechol 2,3-dioxygenase (K00446), 3-oxoadipate CoA-transferase, alpha subunit (K01031), 3-oxoadipate enol-lactonase (K01055), and other enzymes (Figure 5). These functional enzymes are commonly reported to be involved in PAH degradation and petroleum contamination [73]. Simultaneously, the metabolism of xenobiotics by the cytochrome P450 pathway was also annotated (Supplementary Figure S5), indicating that bacterial communities in this study may metabolically degrade exogenous toxic organics such as benzo[a]pyrene, aflatoxin B1, trichloroethene, and 7, 12-dimethylbenz[a]anthracene through cytochrome P450.

In order to illustrate the interaction of various bacteria in the PHE degradation pathway, because each gene annotated in the degradation pathway was associated with each corresponding bacterial species (family level), the contribution of bacteria from different families, such as *Pseudomonadaceae*, *Micrococcaceae*, *Caulobacteraceae*, *Methylobacteriaceae*, *Moraxellaceae*, *Erwiniaceae*, *Oxalobacteraceae*, *Xanthomonadaceae*, and *Propionibacteriaceae*, to genes was studied. As shown in Figure 5, *Pseudomonadaceae* obviously had the highest gene contribution to the pathway, particularly K00152, K14584, K14583, and K14585, which indicates that *Pseudomonadaceae* is indeed a PAH-degrading bacterium with great potential, also supporting the results of microbial community structure analysis. Genes involved in the oxidative transformation of salicylaldehyde into salicylate belong to the *Pseudomonadaceae* family. Then, salicylate is converted into cis,cis-muconate and 2-hydroxymuconate semialdehyde through the ortho-cleavage and meta-cleavage pathways, respectively. The bacteria involved in the downstream metabolic pathway are classified as more diversified and are more closely related to each other, relying on the interaction of several bacteria, such as *Micrococcaceae*, *Rhizobiaceae*, *Hyphomicrobiaceae*, and *Moraxellaceae*, to transform 3-oxoadipate into 3-oxoadipyl-CoA, which is also a sign of entering the TCA cycle. PHE degradation bursts into the naphthalene pathway under the influence of *Pseudomonadaceae*, *Rhizobiaceae*, *Methylobacteriaceae*, *Hyphomicrobiaceae*, and phthalate pathways by *Microbacteriaceae*. Different bacteria perform different functions in PHE degradation. In conclusion, cooperation among microorganisms is beneficial for the degradation of PHE, which may have been the reason for the high degradation rate of PHE in this study. In addition, this study is of guiding significance for the remediation of PAHs at actual sites by studying which microorganisms dominate the degradation of PAHs at different stages.

## 4. Conclusions

In this study, the microbial community with a high efficiency of degrading one PHE was enriched in oil extraction soil, which could degrade 500 mg/L PHE on the fourth day. The microbial community was observed and studied using 16S rRNA sequencing and metagenomic analysis. Studies showed that the main phyla in the microbial community were *Proteobacteria*, *Actinobacteria*, *Firmicutes*, *Bacteroidetes*, and *Fusobacteria*. The function and relative abundance of genes were annotated using the eggNOG, CAZy, and KEGG databases to explain the reasons for the high degradation ability of bacterial communities. Lastly, the functional genes of degradation were correlated with bacteria, and the dominant bacteria in different processes of the degradation pathway were studied to provide guidance for the remediation of actual sites.

## Data Availability

All data analyzed are available from the corresponding author on reasonable request.

## Supplementary Materials

The following supporting information can be downloaded at: https://www.mdpi.com/article/10.3390/ijerph191912039/s1, Figure S1: The metabolic functions of bacterial community based on KEGG database: level 1; Figure S2: The metabolic functions of bacterial community based on KEGG database: level 2; Figure S3: The reconstruction of PHE metabolic pathway. Red arrows represent genes that are enriched, black arrows represent genes that are not enriched, solid arrows represent continuous reactions, and dotted arrows represent two or more reactions; Figure S4: The pathway of Metabolism of xenobiotics by cytochrome P450; Table S1: Ingredients of Minimal Salt Medium; Table S2: Alpha Diversity index for six simples at 97% consistency threshold; Table S3: Functional category of the abbreviation in Figure 2a.
