# Supplementary material for: Metagenomic Analysis of Microbial Alliances for Efficient Degradation of PHE: Microbial Community Structure and Reconstruction of Metabolic Network
Source: Int J Environ Res Public Health. 2022 Sep 23;19(19):12039. doi: 10.3390/ijerph191912039 (PMC9565075; doi:10.3390/ijerph191912039)
Supplement: Supplementary file 1 [file ijerph-19-12039-s001.zip › ijerph-1839456-supplementary.pdf]

The ingredients of Minimal Salt Medium(MSM) which was purchased from coolaber, Beijing, are as follows.

Table S1 Ingredients of Minimal Salt Medium

Ingredients	Content(mg/L)
Na_2HPO_4	2800
NaH_2PO_4	1000
$(NH_4)_2SO_4$	500
$MgCl_2$	53
EDTA	0.5
$FeSO_4 \cdot 7H_2O$	0.2
$CoCl_2 \cdot 6H_2O$	0.02
H_3BO_3	0.03
$CuCl_2 \bullet 2H_2O$	0.001
$NiCl_2 \cdot 6H_2O$	0.002
$Na_2MoO_4 \cdot 2H_2O$	0.003
$CaCl_2 \cdot 4H_2O$	50
$MnCl_2 \bullet 4H_2O$	0.003

Table S2 Alpha Diversity index for six simples at 97% consistency threshold

Sample name	shannon	simpson	chao1	ACE	coverage
F100.3	3.449	0.842	180.917	191.076	0.999
F250.3	3.778	0.868	248.023	262.176	0.999
F500.3	3.622	0.860	195.714	196.038	0.999
F100.6	4.208	0.915	209.279	212.540	1.000
F250.6	3.577	0.836	290.059	296.214	0.999
F500.6	3.556	0.846	303.000	310.802	0.999

Table S3 Functional category of the abbreviation in Figure 2a.

Abbreviation of Functional Category	Functional Category
S	Function unknown
E	Amino acid transport and metabolism
C	Energy production and conversion
T	Signal transduction mechanisms
K	Transcription
P	Inorganic ion transport and metabolism
M	Cell wall/membrane/envelope biogenesis
G	Carbohydrate transport and metabolism
L	Replication, recombination and repair
J	Translation, ribosomal structure and biogenesis
O	Posttranslational modification, protein turnover, chaperones
I	Lipid transport and metabolism
H	Coenzyme transport and metabolism
Q	Secondary metabolites biosynthesis, transport and catabolism
F	Nucleotide transport and metabolism
U	Intracellular trafficking, secretion, and vesicular transport
N	Cell motility
V	Defense mechanisms
D	Cell cycle control, cell division, chromosome partitioning
B	Chromatin structure and dynamics
A	RNA processing and modification
Z	Cytoskeleton
W	Extracellular structures

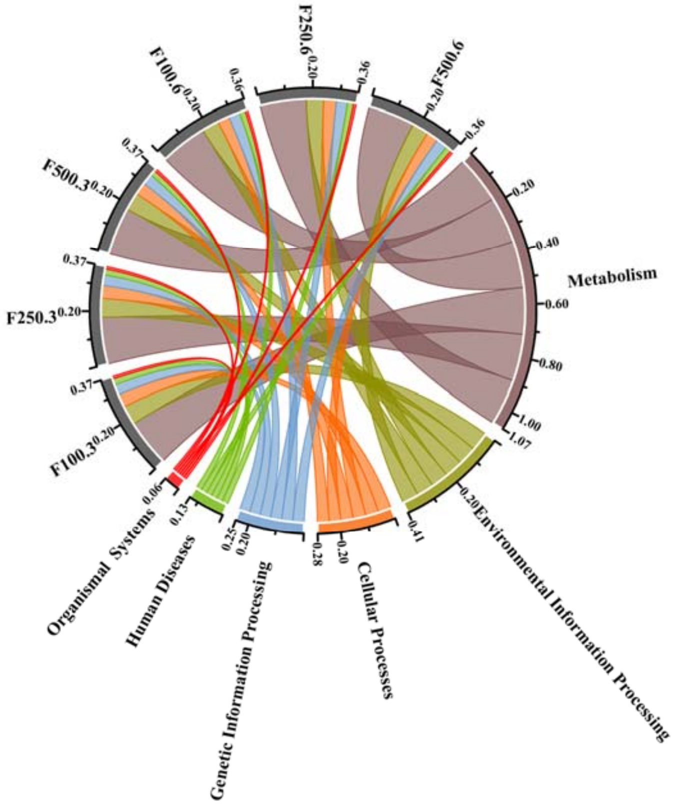

Figure S1 The metabolic functions of bacterial community based on KEGG database: level 1

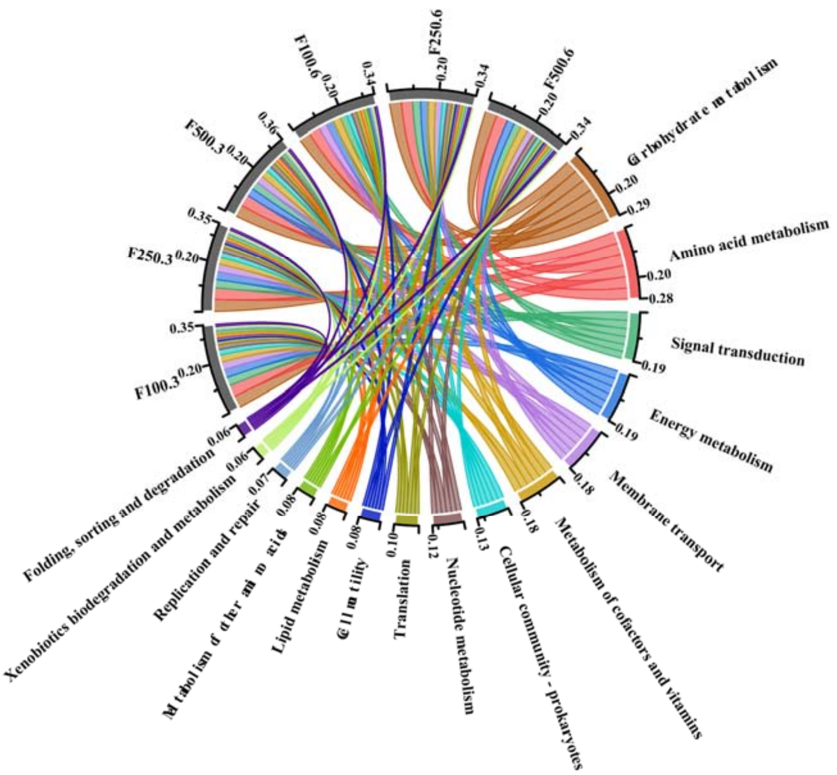

Figure S2 The metabolic functions of bacterial community based on KEGG database: level 2

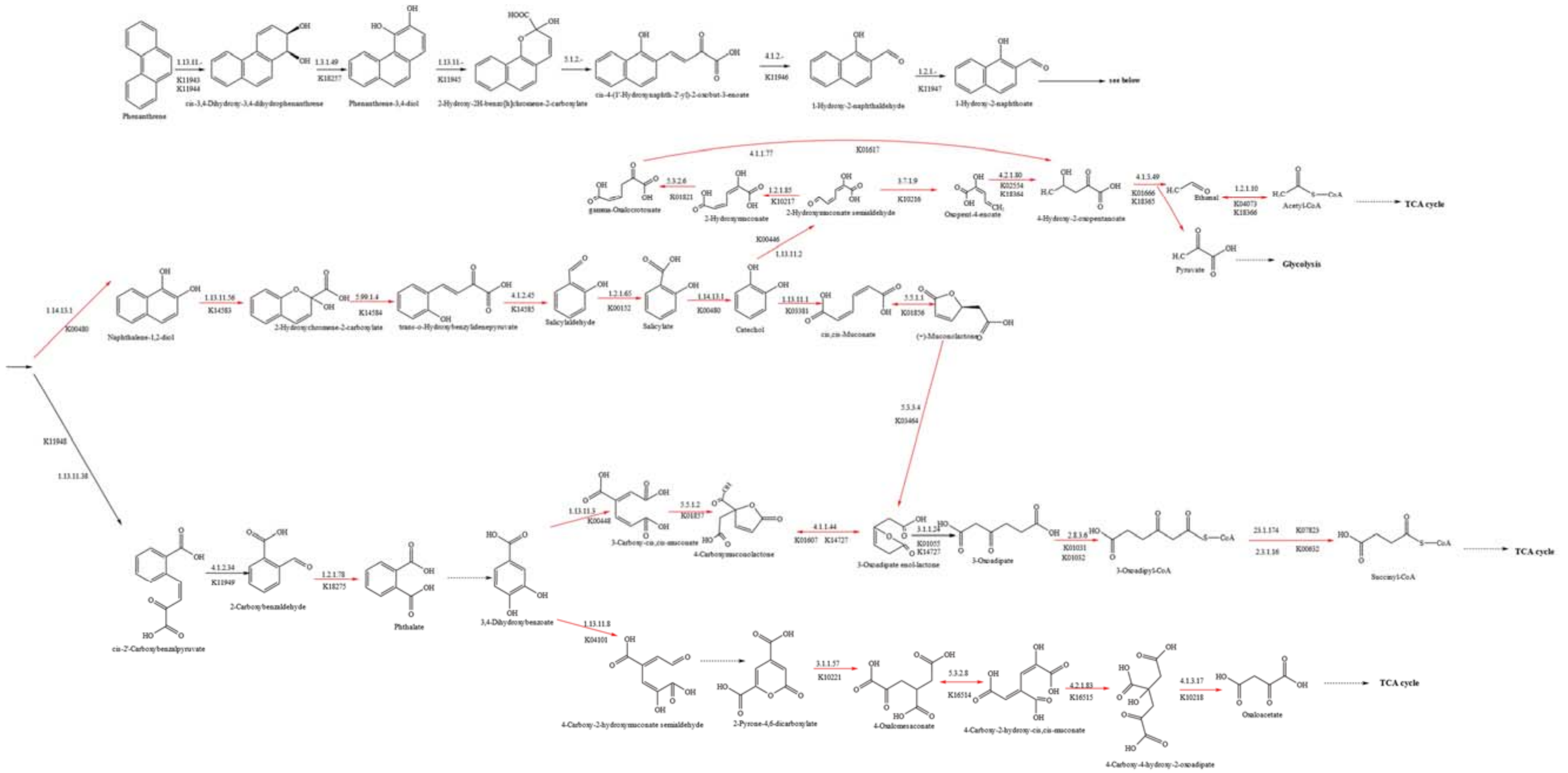

Figure S3 The reconstruction of PHE metabolic pathway. Red arrows represent genes that are enriched, black arrows represent genes that are not enriched, solid arrows represent continuous reactions, and dotted arrows represent two or more reactions.

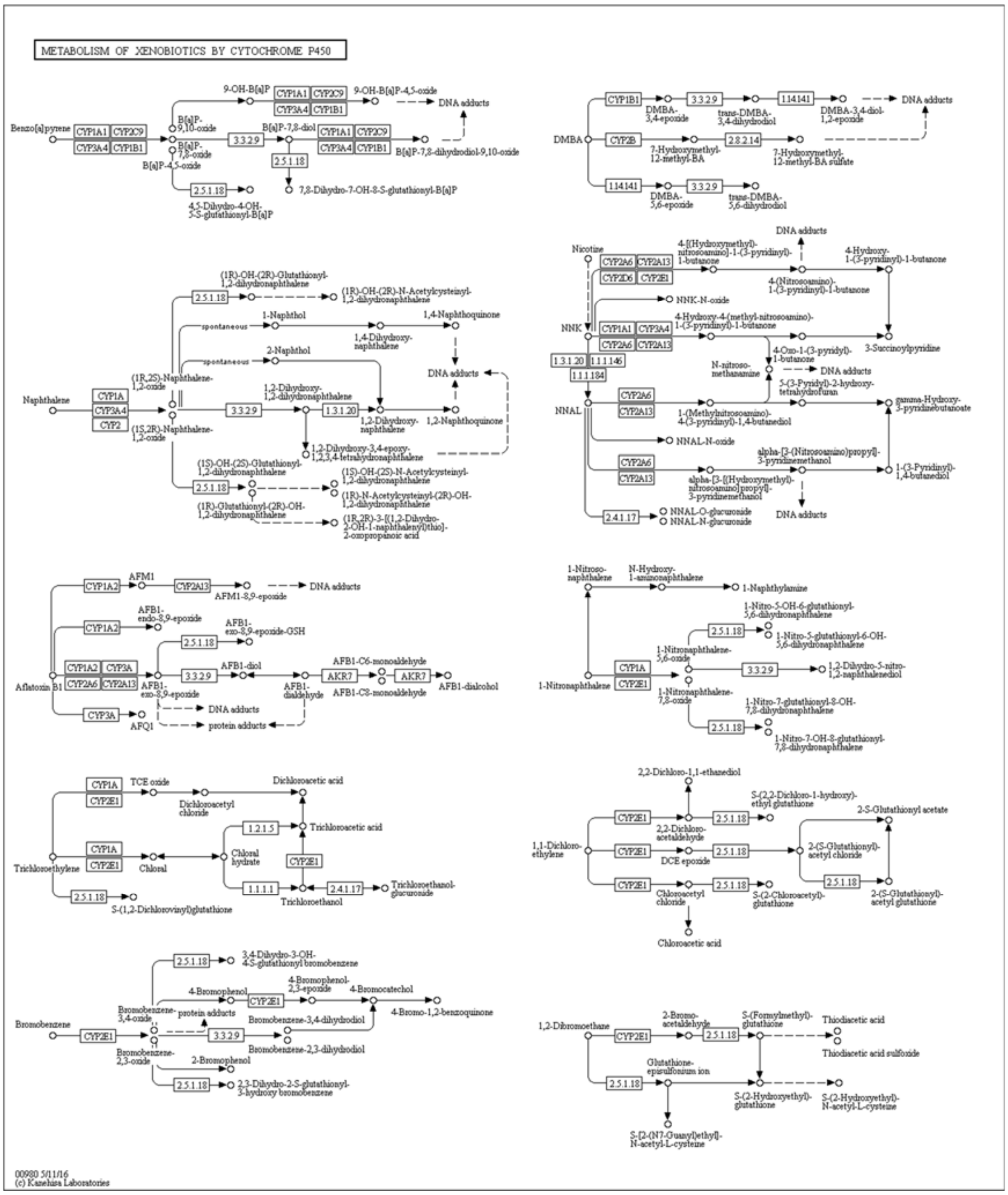

Figure S4 The pathway of Metabolism of xenobiotics by cytochrome P450
